# *JAK2* p.(V617F) mutation in Tunisian myeloproliferative neoplasms and its genotype-phenotype correlation

**DOI:** 10.11604/pamj.2021.39.194.28307

**Published:** 2021-07-12

**Authors:** Hela Sassi, Samia Menif, Safa Ben Ammar, Ahlem Farrah, Hend Ben Hadj Othmen, Hassiba Amouri

**Affiliations:** 1 Laboratory of Molecular and Cellular Hematology, Pasteur Institute of Tunis, University Tunis El Manar, 1002, Tunis, Tunisia,; 2 Faculty of Medicine of Tunis, University Tunis El Manar, 1006, Tunis, Tunisia

**Keywords:** Myeloproliferative neoplasms, polycythemia vera, essential thrombocythemia, primary myelofibrosis, *JAK2* p.(V617F)

## Abstract

Myeloproliferative neoplasms (MPNs) comprise polycythemia vera (PV), essential thrombocythemia (ET) and primary myelofibrosis (PMF). The relationship between JAK2 p.(V617F) mutation and MPNs was first described in 2005. The purpose of this study was to determine the prevalence of JAK2 p.(V617F) mutation in Tunisian patients assessed for MPNs and try to set a genotype-phenotype correlation. A retrospective study was conducted between January 2015 and April 2019. We collected the clinical data of all patients with MPNs suspicion or atypical splanchnic vein thrombosis (SVT). JAK2 p.(V617F) mutation was detected by allele specific real-time quantitative fluorescence PCR (AS-qPCR). We gathered 974 patients who underwent molecular analysis, 55.5% of them were male and 44.5% were female. The median age of all studied patients was 56 years. JAK2 p.(V617F) was found in 349 (35.8%) of total enrolled cases. It was reported in 44%, 37%, 29% and 25% of all patients diagnosed as having respectively ET, PV, PMF and atypical SVT. JAK2 p.(V617F) was negative in 62.2% of patients addressed for suspicion of PV. There was a significant positive correlation between the JAK2 p.(V617F) mutation status, age, gender, white blood cell counts and platelet counts. To our best knowledge, this is the first vast investigation of JAK2 p.(V617F) variant in Tunisia and North Africa with the lowest mutation rate in entire cohort and MPNs subgroups, underlying a specific presentation of this mutation. It is considered as an essential marker of MPNs’ diagnosis and prognosis and is associated with differences in the phenotype of these disorders, helpful for the follow-up of these patients.

## Introduction

Myeloproliferative neoplasms (MPNs) are a heterogeneous group of disorders which arise from genetically altered myeloid stem or progenitor cells. MPNs are characterized by an over production of mature myeloid cells. Classical MPNs have been classified into 3 entities: polycythemia vera (PV), essential thrombocythemia (ET) and primary myelofibrosis (PMF) [1]. It has long been recognized that these diseases are characterized by shared clinical, pathological and molecular features. All MPNs entities arise from a single somatically mutated stem cell that clonally expands and results in a single or multilineage hyperplasia.

The year 2005 was a watershed in understanding of the molecular pathogenesis of MPNs with the identification of an acquired recurrent somatic mutation in the Jak homology domain 2(JH2) of Janus kinase 2 (*JAK2*) gene in a significant proportion of patients with MPNs [2]. This variant represents single nucleotide substitution of G to T at nucleotide 1849 (c.1849G>T) in exon 14 of *JAK2* gene, resulting in the substitution of Valine to Phenylalanine at codon 617 p.(V617F) [3]. This dominant gain of function mutation affects auto inhibitory JH2 pseudokinase domain of the *JAK2* protein, leading to constitutive activation of *JAK2* and JAK/STAT signaling pathway in the absence of ligands [4]. *JAK2*p.(V617F) is detected in 50-60% of ET and PMF patients and up to 97% of PV patients [5]. Infrequent occurrence of this mutation has been reported in acute leukemia, myelodysplasic syndrome (MDS) and atypical chronic myeloid leukemia [6]. In 2008, the world health organization (WHO) classification recognized the *JAK2*p.(V617F) mutation as a major criterion for the diagnosis of MPNs [7]. To our best knowledge, one Tunisian publication studied *JAK2*p.(V617F) mutations in a cohort of 45 patients [8]. The aim of this study is to report the prevalence of *JAK2*p.(V617F) mutation in MPNs Tunisian patients and try to set a genotype-phenotype correlation.

## Methods

**Ethics statement:** this study was approved by the regional committee for medical and health research ethics of Pasteur Institute of Tunis. Written informed consents were obtained from each patient for genetic analysis and publication. No commercial support was involved in this study. All data analyzed in this study are included in this article.

**Study population:** a retrospective study was conducted in the laboratory of hematology of Pasteur Institute of Tunis between January 2015 and April 2019. We included all the patients with clinical suspicion of MPN or atypical splanchnic vein thrombosis for whom *JAK2* p.(V617F) was prescribed by a clinical hematologist. We gathered clinical information in the prescription of all screened patients: age, gender and the applied classification and diagnostic criteria proposed by the World Health Organization (WHO) in 2008 and the updated version 2016 relative to MPNs [9]. We excluded patients with incomplete clinical data.

**Samples´ collection:** blood samples were obtained after written informed consents from all included patients. Blood routine examination involved hemoglobin, white blood cell (WBC) counts and platelet counts.

***JAK2* p.(V617F) mutation detection:** DNA extraction was carried out from lymphocytes by QIAMP DNA mini-Kit (QIAGEN, Germany) according to the manufacturer´s protocol. DNA quality was assessed with the Nanodrop 2000 spectrophotometer (Thermo Fisher Scientific, Waltham, MA, USA) with an approved DNA concentration ranging between 100-200 ng/µL and A260/A280 ratio between 1.8-2.

We performed an Allele specific real-time quantitative fluorescence PCR (AS-qPCR) using ipsogen *JAK2* MutaSearch Kit, designed for the specific and quantitative determination of DNA copy number of *JAK2*p.(V617F) mutation and *JAK2* wild type with respective primers [10]. p.(V617F) positive control (PC-V617F *JAK2*), negative control (NC-V617F *JAK2*), cut-off sample (COS-V617F *JAK2*) were provided by the kit. A 96-well plate qPCR device were used and all measures were analysed in duplicate. For one reaction, the mix contained 12.5 μL of TaqMan Universal PCR Master Mix, 1μL of specific primers and probe and 6.5 μL of H2O. Five μL of DNA samples were added to 20 μL of the corresponding qPCR premix well (V617F or WT). Five μL of each control and COS-V617F *JAK2* were added in each corresponding 20 μL qPCR premix well. The following cycling conditions were run in the 7500 Sequence Detection System (ABI, Melbourne, Australia) to amplify in real time the increase in fluorescence generated: One cycle at 50°C for 2 minutes of incubation, followed by a 10-minute incubation at 95°C, then 50 cycles at 95°C (15 seconds), and an extension step at 63°C (1 minute). The point at which the fluorescent signal rises above the background called crossing threshold (CT), is directly proportional to the quantity of starting DNA. The average CT value obtained (CT V617F and CT WT) for each sample (controls, cut-off sample and patients samples) were calculated. The load limit verified that the patient DNA sample used for the test has been correctly manipulated to ensure the reliability of the final results for *JAK2* V617F status. Then we calculated the ΔCT value for all valid samples (ΔCT Sample) and controls (ΔCT PC- V617F, ΔCT NC- V617F, and ΔCT COS) in order to obtain the ΔΔCT value for each patient sample (ΔΔCT Sample) and each control (ΔΔCT PC- V617F and ΔΔCT NC- V617F). The *JAK2* V617F mutation was detected if ΔΔCT Sample > + ΔCT COS x 0.07 and was absent if ΔΔCT Sample < - ΔCT COS x 0.07.

**Statistical analysis:** descriptive and comparative statistical analysis were performed using the statistical package SPSS for windows, version 16. The tests used for comparison of qualitative variables were Chi-squared test (χ2 test). The tests used for comparison of distributions of quantitative variables were Student's T test and Mann-Whitney U test. The degree of significance “p” retained for all the tests was 0.05.

**Ethics in publishing:** this study was approved by the regional committee for medical and health research ethics of Pasteur Institute of Tunis.

## Results

**Population basic characteristics:** we tested 974 Tunisian patients for *JAK2*p.(V617F) mutation with clinical suspicion of MPN or a typical splanchnic vein thrombosis. The median age of all studied patients was 56 years with extremes ranging from 1 to 90 years and M/F sex-ratio was 541/433. From the total studied population, the MPNs were divided into: 29.4% (286/974) PV, 28.2% (275/974) ET and 9.2% (89/974) PMF. Unclassified MPNs were found in 25.5 % (249/974) of all cases and 7,7% (75/974) patients were addressed for SVT. We did not report any secondary polycythemia. The clinical and biological characteristics in all tested Tunisian patients are summarized in Table 1.

**Table 1:** basic clinical and hematological characteristics in negative and positive *JAK2*p.(V617F) mutation in all tested Tunisian patients

Variables	All cohort (n=974)	*JAK2*p.(V617F) negative (n=625) (A)	*JAK2*p.(V617F) positive (n= 349) (B)	p value A vs B
Mean Age (years)	53	49	59	0.000*
Sex ratio H/F	1.24	1.49	0.91	0.000**
Mean white blood cell count (x10^9^/l)	14.6	14.4	15.7	0.000*
Mean platelet count (x10^9^/l)	632	560.4	742.2	0.000*
Mean hemoglobin (g/dl)	14.6	15.0	15.8	0.150*

(*) Mann-Whitney U test. (**) χ2 test

**Figure 1:**
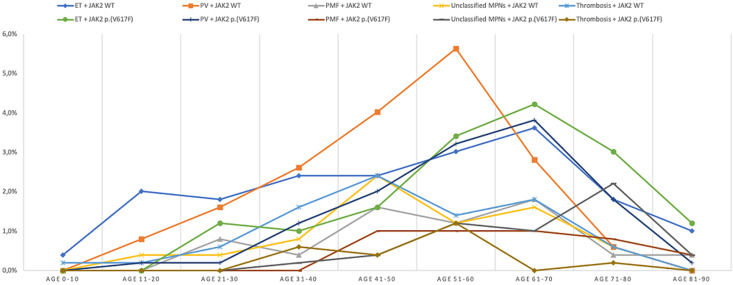
age distribution in wild type and mutant *JAK2* subgroups (ET: essential thrombocythemia; MPNs: myeloproliferative neoplasms; PMF: Primary myelofibrosis; PV: polycythemia vera; WT: wildtype)

**Genotype-phenotype correlation:***JAK2*p.(V617F) mutation was detected in 349 of all 974 patients (35.8 %). *JAK2* p.(V617F) was found in 44% (121/275), 37.8% (108/286), 30.1% (75/249), 29.2% (26/89) and 25.3% (19/75) of patients diagnosed with ET, PV, Unclassified MPNs, PMF and thrombosis respectively. The SVT subgroup were carriers of *JAK2*p.(V617F) mutation despite the absence of overts signs of MPNs. We compared the subgroup with mutant variant to wild type subgroup and found a statistically significant difference in the distribution of age and sex ratio (p= 0.000) (Table 1). Mean age was significantly higher with *JAK2*p.(V617F) mutation in total cohort, three subgroups of MPNs and unclassified MPNs (p=0.000-0.032) (Table 1, Table 2) except in SVT cohort (p=0.856). Age distribution depending on the status of *JAK2* in the different subgroups is represented on Figure 1.

**Table 2:** genotype-phenotype correlation in the different subgroups of myeloproliferative neoplasms in Tunisia

MPNs	Variables	*JAK2*p.(V617F) negative (n=625) (A)	*JAK2*p.(V617F) positive (n= 349) (B)	p value A vs B
**PV (n=286)**	**Mean Age (years)**	49	58	0.000#
	**Sex ratio H/F**	7	1.24	0.000**
	**Mean white blood cell count (x10^9^/l)**	12.107	13.744	0.538#
	**Mean platelet count (x10^9^/l)**	262.985	491.487	0.000*
	**Mean hemoglobin (g/dl)**	18.14	18.06	0.894#
**ET (n=275)**	**Mean Age (years)**	49	60	0.001*
	**Sex ratio H/F**	0.6	0.74	0.374**
	**Mean white blood cell count (x10^9^/l)**	12.713	13.936	0.594 #
	**Mean platelet count (x10^9^/l)**	986.743	994.444	0.899#
	**Mean hemoglobin (g/dl)**	12.39	14.11	0.118#
**PMF (n=89)**	**Mean Age (years)**	55	64	0.032 #
	**Sex ratio H/F**	1.3	0.85	0.376**
	**Mean white blood cell count (x10^9^/l)**	12.399	16.867	0.348 #
	**Mean platelet count (x10^9^/l)**	261.520	304.546	0.703 #
	**Mean hemoglobin (g/dl)**	11.4	9.24	0.594 #

ET: essential thrombocythemia; MPNs: myeloproliferative neoplasms; PMF: Primary myelofibrosis; PV: polycythemia vera. (*) Mann-Whitney U test. (**) χ2 test. (#) Student's T test

**Figure 2:**
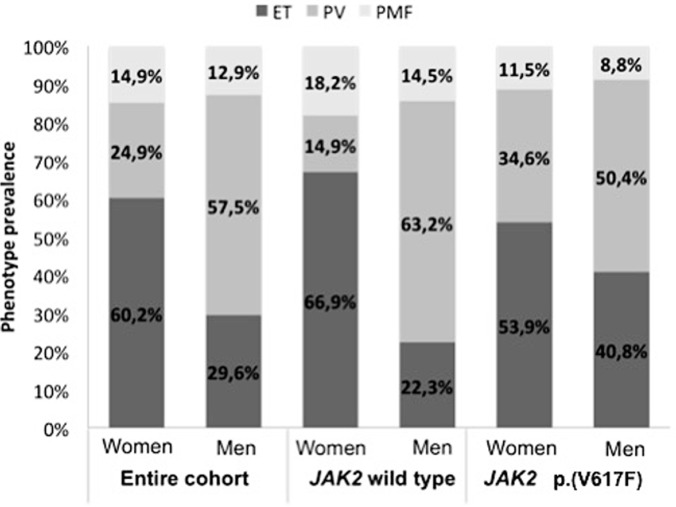
sex-specific differences in Tunisian cohort and wild type or mutant *JAK2* subgroups (ET: essential thrombocythemia; PMF: primary myelofibrosis; PV: polycythemia vera)

Men were more concerned with PV (p<0.001) than women who were more prevalent with PMF clinical phenotype (14.9% vs 12.9%; p<0.001) and ET phenotype (60.2% vs 29.6%; p<0.001) (Figure 2). In *JAK2* wild type subgroup, the sex presentation was significant (p<0.001) where PV phenotype (63.2% vs 14.9%) was more common in men than women whereas ET (66.9% vs 22.3%) and PMF (18.2% vs 14.5%) phenotypes were more reported in women than men (Figure 2). In patients bearing *JAK2*p.(V617F), the sex repartition was not significant (p= 0,2-0.24): women were more common to have ET (53,9% vs 40.8%) and PMF (11.5% vs 8.8%) phenotypes while men tend to have more PV phenotype (50.4% vs 34.6%) (Figure 2). *JAK2*p.(V617F) was associated with a meaningfully higher level in WBC and platelet counts (p= 0.000). There was no significant correlation between the *JAK2* mutational status and hemoglobin levels (p=0.150) (Table 1).

In PV cohort, we noticed that patients with *JAK2*p.(V617F) had significantly higher mean age (p=0.000) and higher platelet counts (p=0.000) than in wild type group. Men were more concerned with *JAK2* wild type (p=0.000) (Table 2). In ET, PMF and SVT subgroups, the mutational status showed no significant correlation with gender, mean levels of hemoglobin, WBC and platelet counts (Table 2). The mutant status in unclassified MPNs presented a higher level of hemoglobin, statistically significant (p=0.007).

## Discussion

In the present study, we determined the prevalence of *JAK2* p.(V617F) mutation in the first large Tunisian patients cohort examined for suspected MPNs or SVT. We also investigated the relationship between this mutation and its influence in hemogram variation in 974 patients divided into different subgroups of MPNs.

Myeloproliferative neoplasms mainly occur in adults and only infrequently in children [11]. Patients with MPNs experience a chronic disease with an insidious onset that often lasts many years, during which they may variably progress to acute leukemia or bone marrow failure [12]. The identification of *JAK2* p.(V617F) mutation is central to the diagnosis of a clonal process and exclusion of non-neoplastic disorders [13]. In 2008, WHO classification mentioned the positivity of the recurrent somatic mutation in exon 14 of *JAK2* gene, as a major criterion of MPNs´ diagnosis specifically ET, PV and PMF. It is detected in around 50-60% of ET and PMF patients and in more than 95% of patients with PV [14].

In this Tunisian study, *JAK2* p.(V617F) mutation was detected in around 35.8 % of all enrolled patients, 44% of ET, decreasing the distribution of this mutation between PV and ET accounting for 37.8% of PV and 29.2% of PMF. It is striking that as many as 62% of those with PV were *JAK2* p.(V617F) negative [15-27] (Table 3), as this was inconsistent with findings in international literature where less than 3% was reported [28]. We managed to compare these rates with large studies (n>200) of different populations ethnicity. Interestingly, to our best knowledge, we found that Tunisian patients have the lowest mutation rate of *JAK2* p.(V617F) in all cohorts and their different subgroups of MPNs [15-27] (Table 3). This could be explained by different sizes of cohorts, several ethnics, single nucleotide polymorphism or environment factors [29].

**Table 3:** associations of *JAK2* p.(V617F) mutation with hemogram variations in different subgroups of myeloproliferative neoplasms in some reported studies and Tunisian patients

Study	Population	All cohort					PV group					ET group					PMF group				
		M	MA	WBC	PC	Hb,	M	MA +	WBC+	PC+	Hb+	M	MA+	WBC+	PC+	Hb+	M	MA+	WBC+	PC+	Hb+
Soliman *et al*. 2020 [15]	Egyptian (n=200)	44%	-	-	-	-	48.9%	51.9	12.8	457	17.9	44.1%	49.9	13.5	1003	13.4	32.5%	57.9	28.8	391	12.6
Syeed 2019 [17]	Indian (n=90)	97.8%	52	-	638.3	16.3	100%	53.5	-	607.5	18.5	-	-	-	-	-	92.3%	50	-	669	14
Misawa *et al*. 2018 [16]	Japanese (n=493)	72.4%	66.4	10.6	547.6	13.4	91.6%	64	14.2	565	18.8	59.9%	67	9.8	847	14.3	53.8%	71	9.7	293	10.4
Lang *et al*. 2018 [18]	Chinese (n=492)	48.2%	51.8	7.8	701.9	16	61.5%	57.4	15.8	518.6	18.8	40,4%	58.5	18.7	929.8	14.7	44.2%	52.4	3.5	166.4	13.7
Rabade *et al*. 2018 [19]	Indian (n=130)	60%	50	12.9	344	11.9	100%	50	11.2	320	18.2	61.7%	48	13.6	781	14.7	57.6%	57	20.6	238	10.6
Gángó*et al*. 2018 [20]	Hungarian (n- 652)	56.7%	63	-	-	-	-	-	-	-	-	55.8%	62	10.5	755	13.8	58.6%	67	13.2	346	11.1
Ojeda *et al*. 2017 [21]	Argentinean(n=439)	74.9%	-	-	-	-	94.9%	-	-	-		61.2%	64	11.3	885	14	62%	64	17	276	11
Singdong *et al*. 2017 [22]	Thailand (n=100)	81%	66.5	24.5	518.7	12.6	94.7%	63	14.9	471.8	17.6	74.5%	62.2	11.7	793.3	12.3	25%	65	13.6	471.5	9.8
Kim *et al*. 2015 [23]	South Korea (n=199)	67.3%	58.3	9.99	584	13.6	87.9%	-	-	-	-	63.3%	57.3	9.8	734	13.5	57.4%	61.9	11	368	11.3
Andrikovic s *et al*.2014 [24]	Hungarian (n- 603)	70.5%	60	-	-	-	100%	61	11	456	18.3	53,3%	61	10	778	14.7	57%	68	12	250	11.6
Ha *et al*. 2012 [25]	South Korea (n=103)	71.8%	64.0	-	-	-	95.5%	62.9	18.9	438.3	18.2	68.8%	66.6	19.1	1111.5	12.9	52.9%	68.7	19.7	440.4	9.3
Sazawal *et al*. 2010 [26]	Indian (n=75)	68%	49.3	19.8	626.5	12.6	82%	53.2	33.6	368	16.2	70%	53	11.7	1000	12.8	52%	49.9	12.6	153	9.8
Benmoussa *et al*. 2009 [27]	Moroccan (n=70)	41.4%	-	-	-	-	89.47%	-	-	-		62.5%	-	-	-		33.3%	-	-	-	
Our cohort	Tunisian (n=974)	35.8%	59	15.67	742.2	15.81	37.8%	58	13.7	491.5	13.7	44%	60	13.9	994.4	14.1	29.2%	64	16.9	304.6	9.24

ET: essential thrombocythemia; Hb: hemoglobin (g/dL); M: Mutation rate of *JAK2* p.(V617F) from each group; MA: mean age in years; MPNs: myeloproliferative neoplasms; PC: platelet count (x10^9^/L); PMF: Primary myelofibrosis; + PV: polycythemia vera; WBC : white blood cell count (x10^9^/L); (**+**):the numbers mentioned in these categories are related to values reported in mutant status. (-): not available.

Nineteen patients with SVT were positive for *JAK2* p.(V617F) mutation. These results support the previous view that routine screening of this mutation appears to be indicated in the presence of SVT [30]. Many studies focused on the correlation between the *JAK2*p.(V617F) mutant status, age, gender, white blood cell counts and platelet counts in different MPNs groups [15-27] (Table 3). The mean age in 974 enrolled patients and in MPNs groups was comparable with literature publications and patients with *JAK2*p.(V617F) mutation are more elder in MPN groups which is conform to recent reports [15-27] (Table 3). Gender specific presentation was raised. Our results were similar to the recent large study of Karantanos *et al*. (2020) in term of women predominance in ET and men in PV independantly of *JAK2*p.(V617F) status but contrasting in PMF where they found more men related to this phenotype (p=0.001) [31]. In their *JAK2* mutant subgroup, we did not established a correlation while their women present more ET and their men more PMF (p=0.001) [31]. In wild type subgroup, our gender presentation was opposite to their results for PMF phenotype (p=5.042) [31].

In MPNs subgroups, *JAK2* p.(V617F) mutation rate was related with variations in hemoglobins, WBC and platelet counts compared with other populations [15-27] (Table 3). Regarding PV cohort, Tunisians had the lowest hemoglobin rate. WBC counts were comparable to Egyptian and Japanese studies [15, 16]. Average platelet counts was among studies with highest rates [16-18]. In ET subgroup, WBC counts in Tunisians were common to Egyptian and Indian findings [15, 19]. As for PV cohort, same conclusion concerning average platelet counts, close to values of Indian, Egyptian and South Korean studies [15, 25, 26]. Relating to PMF subtype, WBC and platelet averages were within the ranges reported in literature but our series had the lowest hemoglobin level [15-27] (Table 3).

Polycythemia is a collective designation for disorders with elevated haematocrit and a distinction is made between absolute and relative polycythemias [32]. Secondary polycythemia are absolute polycythemias that are often due to hypoxia related to underlying heart and lung disease, which was not reported in this study [33]. ET is characterized by an increased platelet count with a megakaryocytic hyperplasia, whereas PMF is a more heterogeneous disorder both by its clinical and biological characteristics, defined by the presence of bone marrow fibrosis and megakaryocytic hyperplasia [34, 35]. In many cases, a continuous between these disease subtypes can be observed, as documented by the progression of ET and PV to secondary myelofibrosis. Boundaries between these disorders cannot be well established and precise diagnosis at disease onset is often challenging. The use of allele specific real-time quantitative fluorescence PCR has significantly increased our ability to detect small amount of mutated clones [36].

Despite advances in understanding MPNs biology, an important fundamental question of how the same genetic event can be associated with distinct clinical phenotypes remains unresolved, as it is for *JAK2* p.(V617F) mutation. The mutational landscape in MPN is much more complex than initially thought [37]. A combination of multiple genetic mutations and a complex interplay of inherited genetic modifiers are likely to be of substantial importance, followed by gender, age and microenvironment disruption [29]. In our large cohort, the allele specific real-time quantitative fluorescence PCR in *JAK2* p.(V617F) was informative. However, our study suffers from limitations and bias since we only search for *JAK2* p.(V617F). We should consider these data in MPN with caution since *JAK2* mutation in exon 12, MPL and CALR mutations were not investigated and further additional studies should test them in the large proportion of the *JAK2* negative patients.

## Conclusion

From our first large study on Tunisian patients with MPNs and SVT, we have settled the prevalence of *JAK2*p.(V617F) mutation in our population and found the lowest mutation rate of this variant in all broad cohorts. Unbalanced rates in different subgroups of MPNs were highlighted. The allele-specific quantitative real-time fluorescence PCR in *JAK2*p.(V617F) keeps its place in routine. The clinical and biological characteristics established in this study could be helpful for the clinicians during the diagnosis and the follow-up of MPNs patients. Furthermore, we must also refine the mutational spectrum of MPNs in Tunisia with additional studies of others driver mutations.

### What is known about this topic

- Molecular genetics is a cornerstone in the diagnosis and follow-up of MPN subgroups.

### What this study adds

- This is the first large retrospective study from Tunisia that provides an overview on clinical and biological characteristics in JAK2 p.(V617F) carriers addressed for suspected MPNs or SVT;
- The lowest rate in JAK2 p.(V617F) mutation (35.8%) was reported in Tunisia in total cohort with 37.8% in PV, uncommon with previous studies.
